# Clinical Performance of CAD/CAM All-Ceramic Tooth-Supported Fixed Dental Prostheses: A Systematic Review and Meta-Analysis

**DOI:** 10.3390/ma14102672

**Published:** 2021-05-20

**Authors:** Babak Saravi, Andreas Vollmer, Maja Hartmann, Gernot Lang, Ralf-Joachim Kohal, Martin Boeker, Sebastian B. M. Patzelt

**Affiliations:** 1 Medical Center—University of Freiburg, Department of Orthopedics and Trauma Surgery, Faculty of Medicine, University of Freiburg, Hugstetter Street 55, 79106 Freiburg, Germany; gernot.michael.lang@uniklinik-freiburg.de; 2 Department of Oral and Maxillofacial Surgery, University Hospital Heidelberg, Im Neuenheimer Feld 400, 69120 Heidelberg, Germany; andr.vollmer@gmail.com; 3 Private Practice, Kantstraße 10, 60316 Frankfurt am Main, Germany; majahartmann@aol.de; 4 Medical Center—University of Freiburg, Center for Dental Medicine, Department of Prosthetic Dentistry, Faculty of Medicine, University of Freiburg, Hugstetter Street 55, 79106 Freiburg, Germany; ralf.kohal@uniklinik-freiburg.de (R.-J.K.); sebastian@patzelt.dental (S.B.M.P.); 5 Medical Center—University of Freiburg, Institute of Medical Biometry and Medical Statistics, Faculty of Medicine, University of Freiburg, Hugstetter Street 55, 79106 Freiburg, Germany; martin.boeker@imbi.uni-freiburg.de; 6 Private Practice, Am Dorfplatz 3, 78658 Zimmern o.R., Germany

**Keywords:** CAD/CAM, dental restoration, ceramic, all-ceramic, zirconia, lithium disilicate, survival, fixed dental prosthesis

## Abstract

Although CAD/CAM ceramics present a promising alternative to metal-ceramic fixed dental prostheses, little is known about their mid- and long-term clinical performance. This systematic review aims to estimate the survival and success rates and describes the underlying complication characteristics for CAD/CAM tooth-supported zirconia- and lithium disilicate-based fixed dental prostheses (FDPs). We systematically searched MEDLINE and Web of Science to find relevant prospective studies with a follow-up of at least one year. We estimated pooled 1-, 5-, and 10-year survival and success rates by combining the collected data in a Poisson regression model. Descriptive statistics were conducted to evaluate the distribution of failures and complications in the included studies. Risk of bias for the included studies was assessed with an adapted checklist for single-arm trials. Pooled estimated 1-, 5-, and 10-year survival rates ranged from 93.80% to 94.66%, 89.67% to 91.1%, and 79.33% to 82.20%, respectively. The corresponding success rates excluding failures, but including any other types of intervention were 94.53% to 96.77%, 90.89% to 94.62%, and 81.78% to 89.25%. Secondary caries was the most frequent cause of failure, followed by chipping of the veneering. The most common cause of complication excluding failures but requiring intervention was chipping of the veneering. Risk of bias was generally acceptable for the included studies, with seven studies associated with low risk of bias, eight studies with a moderate risk of bias, and three studies with serious risk of bias. The current meta-analysis on CAD/CAM-supported FDPs revealed satisfying survival and success rates for up to 10 years of exposure. More prospective studies focusing on long-term performance are needed to strengthen the evidence currently available in the literature.

## 1. Introduction

In the past decades, metal-ceramic restorations have represented the gold standard in fixed prosthetics in dentistry. Zirconium-ceramic crowns are a good alternative to metal-ceramic crowns. They achieve similar incidence rates for biological complications (e.g., secondary caries) and technical aspects (e.g., loss of retention) and reveal better aesthetic properties [1]. There is a steadily increasing demand for alternatives made of all-ceramic materials. Reasons for this may be a growing awareness of biocompatibility and aesthetics [2]. Systematic reviews could already show promising results for the long-term survival of CAD/CAM fabricated lithium disilicate ceramic restorations [3,4]. Studies with a 10-year follow-up period were able to provide survival rates of 96.5% for monolithic as well as for two-layer disilicate ceramics [5].

Dental ceramics are increasingly finding their way in today’s dentistry. In addition to a high degree of stability and aesthetics, they also demonstrate excellent biocompatibility [6]. All-ceramics is a generic term for a tooth-colored, mineral material used to manufacture dental restorations without a metal base [7]. Compared to metal-ceramic systems, all-ceramics show clear advantages for aesthetic appearance with rich color stability due to light-conducting and light-refracting features [8]. In terms of biocompatibility, they convince with low plaque accumulation on their glazed surfaces [9,10]. This can be explained by the fact that ceramics do not dissolve, even in the electrolyte-containing environment of the acidic oral milieu, and behave entirely neutrally toward other materials. Their biocompatibility is even higher than that of alloys containing highly noble metals [11]. Unlike metals, ceramics have the characteristic of thermal insulation and thus have another fundamental advantage. As this prevents thermic transmission, irritation of the vital tooth can be minimized [12].

Similar to the observed progress in dental ceramics, CAD/CAM is increasingly finding its way into modern dentistry. Particularly, in the processing of ceramic materials, significant progress has been made. CAD stands for ‘computer-aided design’ and describes a virtual design of the restoration. CAM stands for ‘computer-aided manufacturing’ and describes the production of dental restorations by using machine units. This technology has become widespread in dentistry and seems indispensable today [13]. In the first step, the three-dimensional anatomy of the respective tooth is recorded with a scanner [14,15]. The next step is digital processing and subsequent transfer to the milling unit [14]. Three production systems can be distinguished here: (1) chairside, where everything takes place in the dental practice; (2) laboratory production, where the milling takes place in the dental technician’s laboratory; and (3) centralized production in an external milling center [14]. Two scanning methods can be distinguished: direct intraoral scan of the teeth and the extraoral scan on a conventional stone cast [14].

With the help of such systems, it is possible to produce ceramic inlays and crowns within one session as a ‘chairside’ procedure [16].

One of the most obvious advantages is the reduced number of appointments and simplified laboratory work compared to conventional restorations [17].

Digitization also provides a decisive advantage in terms of archiving. All clinical data can be stored electronically and allows the restoration to be remade if damaged without a clinical appointment. Compared to the conventional method, CAD/CAM methods reveal a more accurate and reproducible fabrication process [18,19]. The greater accuracy and operator-independent digitalization of the workflow can result in better esthetic outcomes [20]. Due to the shorter production time and reduced number of consultations, less movement of the tooth is likely to occur. This is crucial for accurate and less traumatic restoration of the tooth [21,22]. For these reasons, it is important to question the current gold standard and examine the results of CAD/CAM-made ceramic restorations.

There is currently little known about the mid-term and long-term performance of CAD/CAM fabricated zirconia- and lithium disilicate-based FDPs. Thus, we aim to summarize the current evidence focusing on this promising therapeutical approach’s survival and success rates.

## 2. Materials and Methods

### 2.1. Data Collection

**Figure 1:**
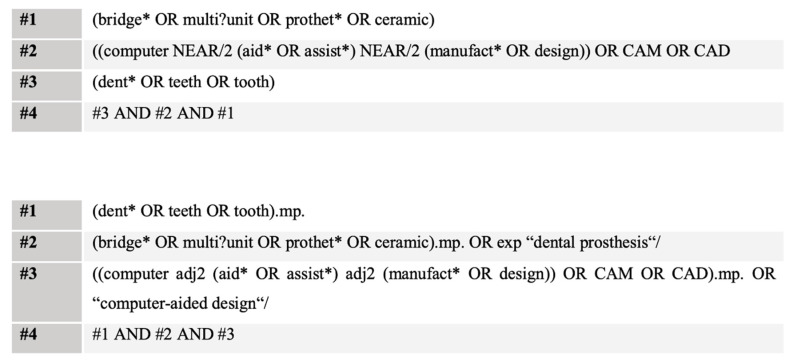
Search strategy.

The present systematic review was performed in accordance with the Preferred Reporting Items for Systematic Reviews and Meta-Analyses (PRISMA) statement [23]. Literature was searched from inception up to January 2020. The target of the structured search approach were prospective studies, focusing on the clinical performance of CAD/CAM fabricated all-ceramic fixed dental prostheses. We applied language restrictions to obtain studies published in English or German. We performed a combined medical-subject heading (MeSH) and free-text term search in Medline (via OVID) and Web of Science core collection (via Web of Science). Furthermore, we hand searched the references of retrieved studies. Truncations were used to retrieve all forms of the search terms. A combination of search terms was performed by the Boolean operators AND and OR (Figure 1). The inclusion criteria were adopted based on a modified PICOS process, taking into account the missing comparator: (P)opulation were human patients with tooth-borne CAD/CAM manufactured all-ceramic fixed dental prostheses, (I)nterventions, in which survival rates and complications as (O)utcomes were assessed within a prospective (S)tudy design.

The following exclusion criteria were defined:

1. Metal and metal-ceramic restorations as an intervention.
2. Implant-borne prostheses as an intervention.
3. Hybrid bridges as an intervention.
4. Cantilever bridges as an intervention.
5. Crowns.
6. Animal studies.
7. In vitro studies.
8. Retrospective studies.
9. Case studies.
10. Languages other than German or English.

Two independent reviewers (M.H. and S.P.) performed the selection of studies in a two-step process. Titles and abstracts were initially screened for relevance, followed by a full-text analysis. Reasons for exclusion were recorded, and any disagreement was resolved by discussion. Data extraction was based on a standardized data extraction form that included all relevant information: study information (author, study design, year of publication, number of patients, and restorations), intervention-related (material type, CAD/CAM information, framework design, luting agent), and outcome-related (follow-up, drop-out, survival data, biological and technical complications, and prognostic factors). Secondary caries, endodontic complications, periodontal pathology, and loss of vitality were considered biological complications, whereas fractures of frameworks or veneering and loss of retention were technical complications.

### 2.2. Assessment of the Risk of Bias

The methodological quality of included studies could not be assessed with the classical Risk Of Bias In Non-randomized Studies of Interventions (ROBINS-I) tool [24] due to the missing comparator (single-arm trials). Hence, a quality assessment tool for the single-arm intervention character of the included studies was adapted based on the checklist developed by Moga et al. [25] and adapted previously [26]. We examined seven domains: (1) Study design, (2) Study population, (3) Intervention and co-intervention, (4) Outcome measures, (5) Statistical analysis, (6) Results and conclusions, and (7) Competing interests and sources of support. The following judgments were performed based on the assessment of these domains: (1) high risk of bias, (2) serious risk of bias, (3) low risk of bias, and (4) no information. Plot visualization was conducted using “robvis” [27].

### 2.3. Statistics

We included all studies matching our predefined inclusion and exclusion criteria in the qualitative synthesis results. Studies reporting the necessary amount of survival data were included in the quantitative results part (meta-analysis). Survival was defined as FDPs were in situ regardless of biological or technical complications. A complication was defined as FDPs that had biological or technical events and were still in situ. Complications were defined according to the United States Public Health Service (USPHS) criteria described by Cvar and Ryge and modified by Wilson et al. [28,29]. The following modified criteria were considered: (1) secondary caries, (2) marginal adaption (marginal integrity), (3) marginal discoloration, (4) loss of anatomical form, (5) surface roughness, (6) color match, (7) endodontic complications, (8) loss of retention, and (9) fracture. The DigitizeIT (http://www.digitizeit.de, accessed on 15 January 2021) software application was used to extract the required quantitative data not reported in the main text of included studies from the Kaplan–Meier curves and graphs. Failure rates (biological and technical failures) were calculated by dividing the number of events (failures) by the total exposure time (in years). Exposure time for each included study was calculated by taking the sum of exposure time for all FDPs. Mean exposure times reported by each included study were applied for statistics if no separate reporting or extractable data was available for individual FDPs. The respective endpoints were last recall, time to failure, and time to drop-out. Exposure times until last conducted recall for drop-out patients were included when possible. Subsequently, the calculated rates were analyzed in a Poisson regression model. The Pearson goodness-of-fit statistics assessed heterogeneity for the model. Three-year, five-year, and 10-year survival proportions were estimated by assuming constant event rates. A *p*-value < 0.05 was considered significant. Means are shown with their standard deviation (sd), whereas median is shown with the interquartile range (IQR). All analyses were performed using Stata Statistical Software Release 15 (StataCorp. 2011, College Station, TX, USA).

## 3. Results

### 3.1. Study Selection and Study Characteristics

**Figure 2:**
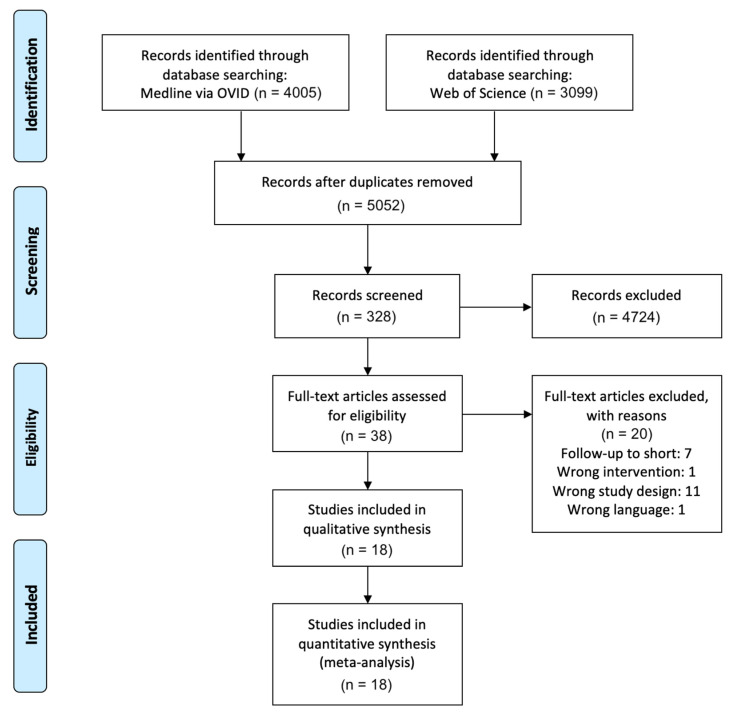
PRISMA flow diagram of the selection process.

A total of 7104 studies were identified through the database search. After removing duplicates and screening the abstracts, 38 studies remained for full-text analysis. Subsequently, 20 studies were excluded with reasons based on full-text analysis, mainly because the predefined study design criteria were not fulfilled (*n* = 11) or the follow-up was shorter than one year (*n* = 7). Finally, 18 publications matching the eligibility criteria could be included in the qualitative synthesis [30,31,32,33,34,35,36,37,38,39,40,41,42,43,44,45,46,47] (Figure 2). All of these studies provided the outcomes with sufficient extractable quantitative data and could be included in the quantitative synthesis.

The study characteristics of the included studies are shown in Table 1. The studies were published between 2005 and 2018. All studies had a prospective design with a median follow-up of five years (range: 1.5–10 years; IQR: 3–9.7 years). One study recruited patients from multiple practices [31], one study in three practices and a university prosthetic department [39], and all other studies were conducted solely in a university institutional environment. One study was a randomized controlled clinical trial focusing on comparing layered versus pressed veneering of 3-unit zirconia-ceramic FDPs [36]. None of the included studies had a controlled or randomized study design comparing the CAD/CAM fabricated all-ceramic FDPs to metal-ceramic FDPs. Overall, 610 patients (range: 15–75) and 669 reconstructions were examined. Available age data of included patients ranged from 19 to 86.4 years. The rate of patients who could not attend the whole observation period (drop-outs) was reported by all studies and ranged from 0% to 21.4%. Eleven studies reported the distribution of maxillary (*n* = 230) and mandibular (*n* = 232) FDPs [30,31,32,34,35,36,38,40,43,45,47]. Seven studies inserted three-unit bridges [32,34,36,38,39,43,45], five studies three- and four-unit bridges [30,31,37,40,42], two studies three-to-five [41,47], and three studies three-to-six-unit bridges [33,44,46]. One study did not provide information [35]. The majority of the studies reported outcomes of posterior FDPs (9/18 studies) or a combination of posterior and anterior FDPs (7/18 studies). One study solely utilized anterior FDPs [44] and one study did not report the examined area [37]. Zircon ceramics were the material of choice in 17/18 studies. One study utilized lithium disilicate (IPS e.max CAD) [39]. The studies utilized eight commercially available CAD/CAM systems: InLab Sirona, Lava System, DigiDent, Cerec Sirona, Cercon (DeguDent), iTero System, Procera System, and DCS System Dentform software in combination with a Precimill machining center. Three studies did not report on the CAD/CAM system [35,41,42]. Most often, restorations were attached by adhesive systems such as RelyX, Panavia, or MultiLink Automix. Five studies utilized glass ionomer cement, whereas two studies used zinc phosphate cement (Table 1). Lops et al. did not provide any information on the cement system [35].

### 3.2. Qualitative Synthesis of Results

Included studies were divided into three groups for the qualitative synthesis of results, depending on the study follow-ups. Group 1 consisted of nine studies with a follow-up of up to approximately four years (mean: 34.6 months; range: 18 to 46 months), group 2 had six studies with a follow-up of up to seven years (mean: 69.3 months; range: 60 to 84 months), and group 3 consisted of studies with a follow-up of up to ten years (mean: 118.2 months; range: 116.4 to 120 months) (Table 2).

**Figure 3:**
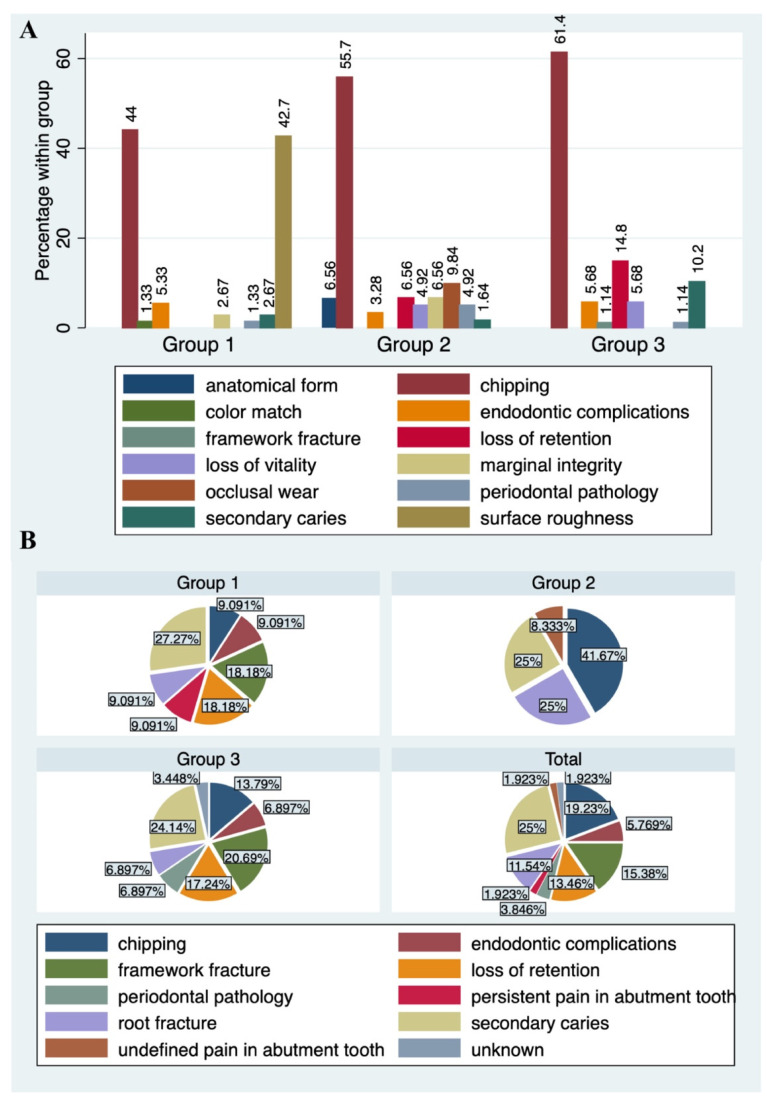
Distribution of complications (**A**) and failures (**B**) stratified by follow-up time frames. Group 1: follow-up from 18 to 46 months; Group 2: follow-up from 60 to 84 months; Group 3: follow-up from 116.4 to 120 months.

Failures were most frequent in Group 3 (failure frequency: 29/186 FDPs; 15.6%). Notably, failure frequencies in Groups 1 (failure rate: 11/268 FDPs; 4.1%) and 2 (failure rate: 12/223 FDPs; 5.4%) were similar. Secondary caries was the leading failure when pooling failures from all three groups (13/52, 25%), followed by chipping (10/52, 19.2%), and framework fracture (8/52, 15.4%). Framework fractures as failures were seen in Group 1 (2/11, 18.2%) and Group 3 (6/29, 20.7%) and were absent in Group 2. This was also the case with endodontic complications as failures. The most frequent failure in Group 2 was chipping (4/29, 13.8%), whereas secondary caries was the leading cause of failure in Groups 1 (3/11, 27.3%) and 3 (7/29, 24.1%). One patient lost his FDP alio loco, and this case was characterized as an “unknown” failure in Group 3. Complications not leading to a removal/replacement of the FDPs were observed in 17/18 studies. Perry et al. was the only study not providing information on complications [37]. The most frequent complication in all groups was chipping of the veneering. It was observed in 44%, 55.7%, and 61.4% of all cases in Groups 1, 2, and 3, respectively. Surface roughness, based on the USPHS criteria, was evident in 42.7% of cases in Group 1. Other cases were not seen in Groups 2 and 3. In fact, this criterion was only mentioned by two studies in Group 1 [36,43], reporting a total of 32 affected cases. Other frequent complications were loss of retention in Groups 2 (6.6% of all group cases) and 3 (14.8% of all group cases) and endodontic complications (Figure 3).

#### 3.2.1. Group 1

The total number of FDPs in Group 1 was *n* = 241 (range: 16–57). Mean follow-up in Group 1 was 34.6 ± 7.9 (range: 18–46 months). Three studies in Group 1 reported survival rates of 93%, 90.5% and 88.4%, respectively [30,39,41]. All other studies in this group did not observe failures in the given study periods. Beuer et al. observed no chipping of veneering porcelain for the 40 months follow-up time frame [30]. A maxillary FDP framework fracture occurred in the mesial abutment after 30-months of service in one FDP. Another mandibular FDP showed loss of retention and was removed. One endodontic treatment was performed due to cold sensitivity. The case was characterized as an “endodontic complication” for statistics as it did not lead to a failure of the FDP. No significant changes were observed for periodontal parameters such as plaque index, bleeding index, and pocket-probing, in the observation period. This led to a survival rate of 90.5% after 40 months. Likewise, Reich et al. observed two failures within 46 months of observation: one technical failure after two years due to a framework fracture in a maxillary ten-unit bridge, and one biological failure caused by a root fracture in a mandibular four-unit bridge. Sailer et al. described two technical failures: chipping of a 4-unit bridge after 38.3 months and one due to loss of retention of a 4-unit bridge after 33.3 months [31]. In contrast, there were five biological failures in the examined cohort. Biological failures included persistent periapical inflammation after 42 months (3-unit bridge), root fracture after 21 months (3-unit bridge), one loss of retention, and finally, three cases of secondary caries after 23.3 months (4-unit bridge), 44.1 months (4-unit bridge), and 33 months (5-unit bridge), respectively. Moreover, eight complications were reported, of which six were chipping of the veneering. Despite this, Naenni et al. did not observe failures [36]. They described a total of 12 chippings of the veneerings within 36 months of service. Furthermore, 18 cases of surface roughness were reported.

#### 3.2.2. Group 2

Group 2 included a total of 208 FDPs (range: 20–59). Follow-ups ranged from 60 to 84 months (mean: 69.3 ± 9.4). Lops et al. reported a survival rate of 88.9% for the 6-year follow-up period [35]. One technical failure occurred due to a framework fracture and one biological failure due to undefined pain in an abutment tooth. In contrast, only a few other complications not leading to removal or replacement of FDPs were observed including two loss of retentions and one chipping case. The workgroup of Sola-Ruiz calculated a survival rate of 88.9% after 84 months [44]. Chipping of the veneering in a six-unit bridge after three years was reported as a technical failure. Biological failures included two cases of secondary caries in a three- and four-unit FDP, respectively, after three years. Two more studies in Group 2 reported chipping as a reason for failures [31,38]. In the cohort of Raigrodski et al., both chipping cases were observed in the posterior mandibular region after 48 and 60 months, respectively [38]. Furthermore, they reported one case of biological failure due to a root fracture of a mandibular second molar, leading to the removal of the 3-unit bridge after 60 months. Furthermore, Burke et al. overall reported eight cases of chipping: five cases occurred in the anterior region, one after one year, and the remaining four after five years of function [31]. Patients were unaware of the chipping, and the cases were polished, remained in service, and were not counted as a failure. In two further cases (pontic tooth 14 after two years and pontic tooth 46 after five years), the repair attempt failed, but the FDPs remained in situ. These cases were also not considered as failures but complications. One chipping case involved the mesial-incisal angle of a central incisor (abutment tooth 11), and the bridge was replaced (failure). Sorrentino et al. did not find failures of FDPs in the 60 month study observation period [45]. However, 16 complications not leading to the removal of FDPs were mentioned. Of these cases, three were chipping of the veneering. Ioannidis et al. reported an 85% survival of FDPs after 6.3 years. Biological events caused all three failures. However, 23 complication cases were counted, of which 16 were chipping of the veneering.

#### 3.2.3. Group 3

A total of 154 FDPs (range: 22–99) FDPs were included in Group 3. Follow-up ranged from 116.4 to 120 months (mean: 118.2 ± 1.4). Rinke et al. revealed a survival rate of 75% for a mean follow-up of 9.9 years [40]. Overall, 24 fatal failures were found, of which 13 were caused by technical and 10 by biological events. One FDP was lost alio loco and was characterized as an “unknown” failure. Furthermore, 50 complications were reported, of which 31/50 (62%) were chipping of the veneering. In contrast, Teichmann et al. reported a higher survival rate of 95% after 120 months of service [46]. The only failure was due to an endodontic complication caused by recurrent pain, subsequent endodontic revision, and finally, apicoectomy. Moreover, nine complications were reported, of which the majority were chipping (8/9, 88.9%). Similarly, Chaar et al. described 93.6% survival after 116.4 months [32]. In addition to the four failures, 29 complications were observed, of which 15 were chipping of the veneering.

### 3.3. Quantitative Synthesis of Results

Estimated failure rates, survival rates, and success rates for the different groups are listed in Table 3. The Pearson goodness-of-fit test to check for heterogeneity and the necessity of the random-effects Poisson regression was not significant for any of the individual groups and rates (*p* > 0.05), and estimates were determined by standard Poisson regression. The estimated rates were similar across the different follow-up groups, with more variation for the estimated success rates, specifically, the 5-year and 10-year success estimates. Estimated 3-year survival rates ranged between 93.80% to 94.66%, 5-year survival rates ranged from 89.67% to 91.1%, and 10-year survival rates from 79.33% to 82.20%. Success rates considered complications with FDPs excluding failures. Estimated 3-year success rates ranged between 94.53% to 96.77%, 5-year success rates from 90.89% to 94.62%, and 10-year success rates from 81.78% to 89.25%.

#### 3.3.1. Group 1

A total of 11 FDPs over a 713.91 years cumulative exposure time have failed in Group 1. The estimated failure rate per 100 FDP years was 1.78 (95% CI: 1.435–2.216). This translates to a 3-year, 5-year, and 10-year survival estimate of 94.66%, 91.1%, and 82.2%, respectively, for Group 1. An estimated complication rate per 100 FDP years of 1.24 (95%CI: 0.932–1.661) was calculated for the *n* = 75 complications. The respective success rates were 96.27%, 93.78%, and 87.56% for 3-, 5-, and 10-years of exposure.

#### 3.3.2. Group 2

The total FDP exposure time in Group 2 was 1208.1 years. Overall, 12 FDPs failed, and 61 showed complications excluding failures. Failure rate per 100 FDP years was estimated to be 2.07 (95% CI: 1.555–2.746). Survival rate dropped from 93.8% for the 3-year-data to an 89.67% 5-year rate, and finally, 79.33% for the 10-year survival rate. A total of 61 complications were part of Group 2, leading to a complication rate per 100 FDP years of 1.08 (0.759–1.523) with respect to the 1208.1 years of total exposure time. The resulting estimated success rates for 3-, 5-, and 10-year of exposure time were 96.77%, 94.62%, and 89.25%, respectively.

#### 3.3.3. Group 3

Group 3 included a total of 29 failures and 1470.65 years of total exposure time for the three studies, leading to an estimated failure rate per 100 FDP years of 1.82 (95%CI: 1.716–1.935). Estimated 3- and 5 years survival rates were 94.53% and 90.89%. A more pronounced drop in survival rates was observed for the 10-year data (81.78%). A total of 88 complications were recorded for the three studies. Similar to the survival data results, estimated success rates dropped from 94.53% for the 3-year data to 90.89% and 81.78% for the 5-year and 10-year data, respectively.

### 3.4. Assessment of the Risk of Bias

**Figure 4:**
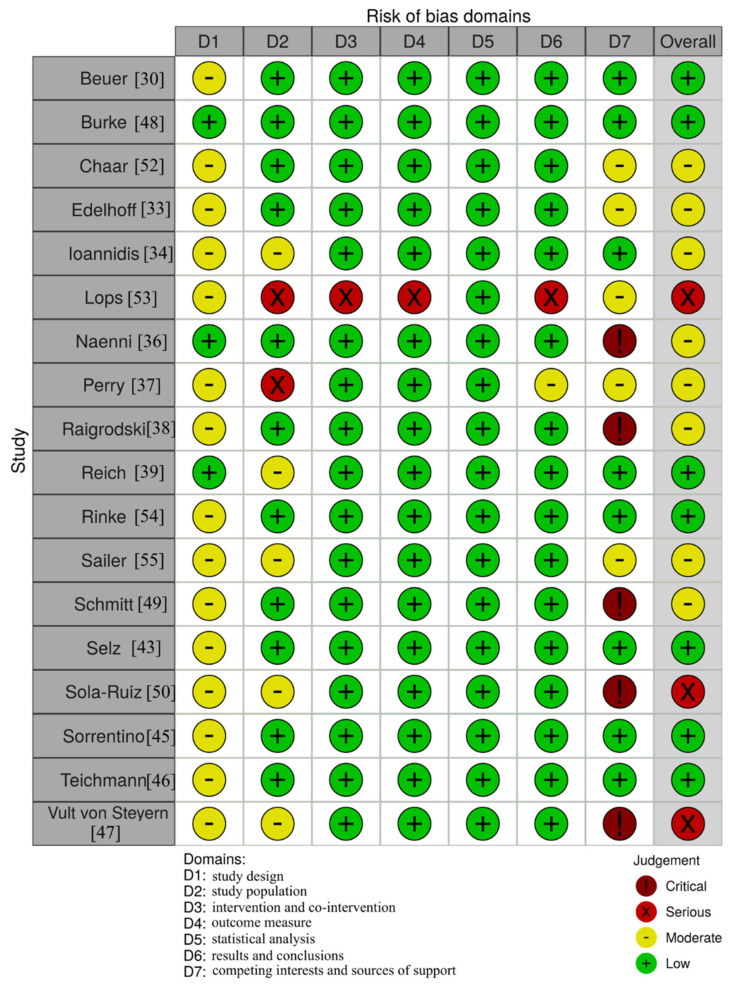
Risk of bias assessment for the included studies.

From the 18 included studies, 7/18 had a low risk of bias, 8/18 had a moderate risk of bias, and 3/18 had a serious risk of bias. Most of the studies were judged to have a moderate risk of bias in the “study design” domain due to not reporting whether patients were included consecutively or not. Five of the studies were judged with “critical risk of bias” in the “competing interest and sources of funding” domain [36,38,42,44,47]. This was based on the fact that these studies provided neither the conflicts of interest statement nor the funding sources. In fact, this was the main cause leading to the overall judgment “serious risk of bias” for Sola-Ruiz et al. [44] and Vult von Steyern et al. [47] because all other domains but D1 were judged to have a low risk of bias. For Naenni et al. [36], the “critical risk of bias” judgment in D7 caused the overall judgment “moderate risk of bias”, even though all other domains were associated with a low risk of bias. The domains D2–D7, focusing on the methodology and results section, were overall associated with a low risk of bias throughout most studies. Only Lops et al. [35] had several domains with the judgment “serious risk of bias” due to lack of information regarding the predefined inclusion/exclusion criteria and other necessary patient-related information such as mean age. Furthermore, Perry et al. [37] were judged with a “serious risk of bias” in the domain “study population” due to not providing sufficient information regarding patients’ baseline characteristics. The overall quality of the included studies was adequate to support the respective studies’ results as assessed with the domain D7 (Figure 4).

## 4. Discussion

The present study aimed to evaluate the clinical performance of CAD/CAM manufactured all-ceramic fixed dental prostheses. The available evidence provided a sufficient amount of data to allow for a satisfying conclusion on the success and survival of such dental therapies. We estimated survival rates of up to 94.66%, 91.1%, and 82.2% for the 3-, 5-, and 10-year survival data based on the available evidence in the literature. The low range variability between the study groups supports the assumption of good accuracy of survival. Similar satisfying results were seen for the success rates focusing on any intervention on FDPs after insertion without considering failures as events. The estimated lowest success rates were up to 96.77%, 94.62%, and 89.25% for the 3-, 5-, and 10-year success data, respectively. Despite the higher variability of success values than survival values between the different follow-up groups for the 5- and 10-year success rate estimates, these values were still in a satisfying area of success.

Notably, the only three studies observing failures in Group 1 had a follow-up period of 3 to 3.8 years [30,31,39] and reported a survival rate of 84.8%, 93%, and 90.5%, respectively. These reported rates were lower than the estimated rates of 93.8% to 94.66% for the 3-year survival based on a meta-analysis of the included studies’ data. Reich et al. (2014) used lithium disilicate ceramics as a base, the other publications mainly used zirconia [39]. Therefore, the survival rates of 93% after 4.7 years described by Reich et al. must be considered critically. Furthermore, FDPs in the premolar region were examined, exposed to lower force effects than FDPs in molar regions [39]. The different mechanical properties of the two ceramic composites were not taken into account here and likely make a direct comparison inaccurate. However, the study’s overall quality was satisfying as assessed with the risk of bias tool, allowing the support of the study results provided for lithium disilicate bases. In contrast, data of the five studies [31,34,35,38,44] in Group 2 reporting survival rates of 85% to 97% for the 5- to 7-year study period were comparable to the 5-year survival estimates of 89.67% to 91.1%, calculated for all studies. This translates to a satisfying correlation and reliability for the 5-year data. The outliner with the highest reported survival rates was Burke et al., providing a survival rate of 97% for the 5-year observation [31]. The least comparable data were found in Group 3, reporting survival rates of 75%, 93.6%, and 95% for the approximately 10 years of observation [32,40,46]. The corresponding estimates calculated from the meta-analysis were lower, with values from 79.33% to 82.2%. In fact, the studies within Group 3 showed high variability in the reported survival rates. The most comparable survival rate was provided by Rinke et al. reporting survival rates of 75% for the 10-year observation period [40]. Overall, there seems to be a more pronounced drop in survival rates between the 5-year and 10-year data. Thus, more data on 10-year survival are warranted to draw a reliable conclusion on the long-term performance of CAD/CAM all-ceramic FDPs.

The most common reason for failure for the pooled included studies was secondary caries. The occurrence of secondary caries has been described by five studies [32,34,40,41,44] involving a total of 26 cases, of which 14 led to a failure of the FDP. Interestingly, a systematic review from Sailer et al. did not find a significant difference in caries incidence for abutment teeth [48]. However, Pjetursson et al. showed a higher caries prevalence on all-ceramic restorations compared to metal-ceramic restorations for multi-unit bridges [49].

The second common reason for failure was chipping. Eight publications showed either chipping or cracks of the ceramic, requiring replacement of the restoration [31,38,44,50]. This accounts for 10/52 (19.2%) of all observed failure cases. In a systematic review, Heintze and Rousson et al. were able to show a significantly higher chipping rate of the veneering ceramic in bridges with zirconia frameworks than in conventional metal-ceramic bridge restorations [2]. No difference was found between 3- and 4-unit bridges. Furthermore, they reported that 24% of all zirconia FDPs examined revealed chipping, which is in accordance with our results, not considering drop-outs (127/603, 21.06%). In comparison, 43% of the metal-ceramic FDPs showed chippings. However, they reported that when comparing only studies that directly compared zirconia and metal-ceramic FDPs, chipping frequency was higher for zirconia FDPs [2]. Sailer et al. (2009) found chipping in 33% of all-ceramic bridges after three years. In comparison, chipping was found in 19.4% of the metal-ceramic restorations [51]. However, they found no difference in terms of survival rates after three years of function [51]. In order to replace metal-ceramic restorations as the gold standard, an improvement in the bond between the veneer and the framework ceramic must be achieved. In vitro studies showed that the leading cause of chipping was lying in the veneering ceramic [52], which can often be treated by polishing. The reasons for chipping can be the different layer thicknesses and the design of the framework as well as different temperature expansion coefficients of the ceramics [30,51]. Further attempts to reduce chipping were investigated by Guess et al. (2010) by comparing monolithic lithium disilicate crowns with veneered zirconia crowns [52]. Monolithic restorations performed better, although further research is needed to be able to assess this conclusively [52]. In summary, it can be concluded that more focus should be set on framework-veneering interfaces and veneering ceramics properties to reduce chipping rates in the future. One way to accomplish this task could be the improvement of the design of the framework itself or strengthening the veneering ceramics.

Regarding framework fractures of all-ceramic restorations, Sulaiman et al. (2020) were able to provide low failure rates of 1.35% for lithium disilicate as well as for monolithic restorations in a follow-up period of up to 7.5 years. Similar to the chipping behavior results, monolithic restorations were also found to be at lower risks for framework fractures [53]. Focusing on CAD/CAM produced all-ceramics, Belli et al. (2016) was able to show equivalent fracture rates of 1.4% over a period of 3.5 years [54].

Loss of retention as another type of complication leading to either failure or repair without replacement of the FDP was addressed by six studies [30,31,32,35,44,50] and occurred in 22/603 (3.65%) of all cases, not considering drop-outs. Five of these studies reported the cementation material and used adhesive [41,44] or conventional [30,32,40] methods. Most of the loss of retention cases were reported by Rinke et al. (12/22, 54.55%) and Chaar et al. (6/22, 27.27%), both using conventional cementing methods [32,40]. Similar results regarding the loss of retention in zirconia frameworks have been described by Tinschert et al. for conventional cementing techniques as well as Sailer et al. for adhesive cementation [41,55].

The searched literature was limited to the medical databases MEDLINE and Web of Science, which was considered to be an acceptable limitation, as most of the international peer-reviewed articles were included. We only included human studies, but did not consider in vitro examination. There might be other results regarding survival rates if in vitro-studies were included. However, we wanted to implement real world data including representative loading situations in the human oral cavity, allowing translation and generalization of outcomes. One possible way of further in-depth ex vivo biological and mechanical testing of CAD/CAM restorations could be the application of post-treatment analysis using a combination of µ-CT and finite element techniques [56,57]. We included only prospective studies, leading to a higher evidence grade compared to the inclusion of case series and retrospective studies. Furthermore, this approach allowed us to compare the data in a meta-analysis reliably. It should be mentioned that none of the publications had a control group with metal-ceramic restorations. The focus of our review was restricted to all-ceramic restorations solely, specifically their clinical performance. Thus, the provided data did not allow for direct comparisons between metal-ceramics and all-ceramics restorations, as this would require an appropriate control group to calculate and compare the necessary effect sizes in a meta-analysis. Notably, patient diseases and medications can affect biological parameters such as periodontal bone quantity, and should be considered in future studies to account for these confounding factors [58,59,60,61]. Another limitation was the small number of studies focusing on long-term survival rates, which should be addressed in future studies. Furthermore, we restricted this review to all-ceramic restorations only. With this in mind, new materials such as polyetheretherketone (PEEK) can be considered in the future. In addition to high-temperature and thermoelastic properties, this material is reported to have a high biocompatibility and abrasion resistance and is already finding applications in dentistry [62,63]. In a recent investigation, PEEK showed a breaking load force of 1283 N and thus qualified as a suitable material for restorations. A recent comparison of hybrid ceramics, zirconia, and PEEK single-tooth crowns showed no significant difference between hybrid ceramics and PEEK [62].

In summary, all-ceramic bridge restorations fabricated by the CAD/CAM procedure and supported by natural abutment teeth are promising prosthetics associated with good short- and long-term clinical survival and success rates. All-ceramic bridge restorations have proven to be a robust framework material with a low fracture susceptibility. They are also a good prosthetic solution for multi-unit bridges in the anterior and posterior regions. Nevertheless, all-ceramic bridge restorations fabricated by the CAD/CAM procedure are associated with specific problems such as technical (e.g., chipping, loss of retention) or biological (e.g., secondary caries) complications, which could be linked to a semi-optimal framework design and/or fit, especially in comparison to metal-ceramic restorations. All-ceramic restorations utilizing CAD/CAM manufacturing will increasingly find their way into daily clinical practice and might lead to a future shift toward all-ceramic therapy options in modern dentistry. Further developments in technology and materials will pave this way. Finally, we encourage authors to provide more data on the long-term performance of CAD/CAM manufactured all-ceramic FDPs.

## 5. Conclusions

CAD/CAM zirconia- and lithium disilicate-based FDPs revealed satisfying survival and success rates for up to 10 years of exposure. Certain associated complications such as chipping and secondary caries were frequently seen in the included studies, and a future in-depth analysis of their underlying factors would be of clinical relevance. More prospective studies focusing on long-term performance are needed to strengthen the evidence currently available in the literature.

## Figures and Tables

**Table 1:** Baseline characteristics of included studies.

Study and Year of Publication	No. of Patients	No. of FDPs	No. of FDP Units	Region (Percentage)	Mean Age/ Range	CAD/CAM	Cement	Drop-Out (%)
Beuer et al. 2009 [30]	19	21	3	posterior	50.9 (27–71)	InLab Sirona	Glass ionomer cement	0
Burke et al. 2013 [31]	36	41	3–4	anterior (33%)/ posterior (67%)	NR	Lava System	RelyX	20
Chaar et al. 2015 [32]	58	65	3	posterior	46.8	InLab Sirona	Glass ionomer cement	9.2
Edelhoff et al. 2008 [33]	18	22	3–6	Anterior (19.1%)/ posterior (80.9%)	19–70	DigiDent	Glass ionomer cement	5.6
Ioannidis et al. 2016 [34]	55	59	3	posterior	52.6	InLab Sirona	Panavia	3.6
Lops et al. 2012 [35]	28	28	NR	anterior/ posterior (NR)	NR	NR	NR	14.3
Naenni et al. 2015 [36]	40	40	3	posterior	52.3	Cerec Sirona/InLab milling unit	Panavia	10
Perry et al. 2012 [37]	15	16	3–4	NR	NR	Lava System	RelyX	0
Raigrodski et al. 2012 [38]	16	20	3	posterior	48 (36–60)	Lava System	RelyX	6.3
Reich et al. 2014 [39]	33	38	3	anterior/ posterior (NR)	54.8 (31.2–86.4)	InLab System/MC XL milling unit (Sirona)	Multilink Automix	3
Rinke et al. 2018 [40]	75	99	3–4	posterior	49.4 (26–76)	Cercon (DeguDent)	Zinc phosphate cement	24.2
Sailer et al. 2006 [31]	45	57	3–5	posterior	NR	NR	Variolink/Panavia	20
Schmitt et al. 2009 [42]	30	30	3–4	posterior	52.2 (27–75)	NR	Glass ionomer cement	10
Selz et al. 2015 [43]	24	24	3	Anterior (8.3%)/ posterior (91.7%)	48.5 (30–65)	iTero System/ 3 + 1 axes milling unit	Multilink Automix	8.3
Solá-Ruiz et al. 2015 [44]	27	27	3–6	anterior	30–65	Lava System	Multilink Automix	0
Sorrentino et al. 2012 [45]	37	48	3	posterior	45.3	Procera System/Procera Center	RelyX	0
Teichmann et al. 2018 [46]	17	22	3–6	Anterior (15%)/ posterior (85%)	40.1	DigiDent	Glass ionomer cement	9.1
Vult von Steyern et al. 2005 [47]	18	20	3–5	Anterior (25%)/ posterior (75%)	NR	DCS Dentform Software/Precimill machining center	Zinc phosphate cement	0

**Table 2:** Survival characteristics of studies stratified by follow-up time frames.

Group 1						
Study and Year of Publication	No. of FDPs after Drop-Outs	Follow-Up in Months (Years)	Type of Material	No. of Failure (Distribution of Failures)	No. of Complications (Distribution of Complications) *	Reported Survival Rate (%)
Edelhoff et al. 2008 [33]	21	39.1 (3.3)	Zirconia with veneering ceramics	0	4 (chipping: 3 periapical pathology: 1)	100
Beuer et al. 2009 [30]	21	40 (3.3)	Zirconia with veneering ceramics	2 (framework fracture:1 loss of retention: 1	1 (endodontic complications: 1)	90.5
Vult von Steyern et al. 2005 [47]	20	24 (2)	Zirconia with veneering ceramics	0	3 (chipping: 3)	100
Perry et al. 2012 [37]	16	24 (2)	Zirconia with veneering ceramics	0	3 (chipping: 2)	100
Selz et al. 2015 [43]	22	18 (1.5)	Zirconia with veneering ceramics	0	19 (chipping: 2 marginal integrity: 2 color match: 1 surface roughness: 14)	100
Sailer et al. 2006 [41]	46	36 (3)	Zirconia with veneering ceramics	7 (secondary caries: 3 loss of retention: 1 chipping: 1 endodontic complications: 1 root fracture: 1)	8 (chipping: 6 secondary caries: 2)	84.8
Naenni et al. 2015 [36]	36	36 (3)	Zirconia with veneering ceramics	0	30 (chipping: 12 surface roughness: 18)	100
Schmitt et al. 2009 [42]	27	34.2 (2.9)	Zirconia with veneering ceramics	0	3 (chipping: 3)	100
Reich et al. 2014 [39]	32	46 (3.8)	Lithium disilicate ceramics	2 (framework fracture: 1 persistent pain in abutment tooth: 1)	5 (chipping: 2 endodontic complications: 3)	93
**Group 2**						
**Study and** **Year of Publication**	**No. of FDPs after Drop-Outs**	**Follow-Up in Months (Years)**	**Type of Material**	**No. of Failure** **(Distribution of Failures)**	**No. of Complications** (Distribution of Complications) *	**Reported** **Survival Rate** **(%)**
Lops et al. 2012 [35]	24	78 (6.5)	Zirconia with veneering ceramics	2 (framework fracture: 1 undefined pain in abutment tooth: 1)	3 (chipping: 1 loss of retention: 2)	88.9
Raigrodski et al. 2012 [38]	19	60 (5)	Zirconia with veneering ceramics	3 (chipping: 2 root fracture: 1)	4 (chipping: 2 marginal integrity: 1 endodontic complications: 1)	90
Sorrentino et al. 2012 [45]	48	60 (5)	Zirconia with veneering ceramics	0	16 (chipping: 3 occlusal wear: 6 marginal integrity: 3 anatomical form: 4)	100
Burke et al. 2013 [31]	33	60 (5)	Zirconia with veneering ceramics	1 (chipping: 1)	7 (chipping: 7)	97
Solá-Ruiz et al. 2015 [44]	27	84 (7)	Zirconia with veneering ceramics	3 (secondary caries: 2 chipping: 1)	8 (chipping: 5 loss of retention: 2 periapical pathology: 1)	88.9
Ioannidis et al. 2016 [34]	57	75.6 (6.3)	Zirconia with veneering ceramics	3 (root fracture: 2 Secondary caries: 1)	23 (chipping: 16 loss of vitality: 3 periodontal pathology: 2 secondary caries: 1 endodontic complications: 1)	85
**Group 3**						
**Study and** **Year of** **Publication**	**No. of** **FDPs after** **Drop-Outs**	**Follow-Up in Months (Years)**	**Type of Material**	**No. of Failure** **(Distribution of** **Failures)**	**No. of Complications** **(Distribution of** Complications) *	**Reported** **Survival Rate** **(%)**
Rinke et al. 2018 [40]	75	119 (9.9)	Zirconia with veneering ceramics	24 (framework fracture: 4 chipping: 4 loss of retention: 5 secondary caries: 6 periodontal pathology: 2 root fracture: 2 unknown: 1)	50 (chipping: 31 framework fracture: 1 loss of retention: 7 secondary caries: 6 loss of vitality: 5)	75
Teichmann et al. 2018 [46]	20	120 (10)	Zirconia with veneering ceramics	1 (endodontic complications: 1)	9 (chipping: 8 periodontal pathology: 1)	95
Chaar et al. 2015 [32]	59	116.4 (9.7)	Zirconia with veneering ceramics	4 (framework fracture: 2 secondary caries: 2	29 (chipping: 15 loss of retention: 6 endodontic complications: 5 secondary caries: 3)	93.6

* Complications were defined according to the modified USPHS criteria and considered as biological or technical complications not leading to a replacement of the bridge (no failures).

**Table 3:** Illustration of estimated failure rates, complication rates, and survival rates stratified by follow-up time frames.

Study and Year of Publication	Total FDP Exposure Time	Total No of Failures	Estimated Failure Rate Per 100 FDP Years (95% CI)	Estimated 3 Year Survival Rate (%)	Estimated 5 Year Survival Rate (%)	Estimated 10 Year Survival Rate (%)	Total No. of Complications *	Estimated Complication Rate (Per 100 FDP Years)	Estimated 3 Year Success Rate	Estimated 5 Year Success Rate	Estimated 10 Year Success Rate
Edelhoff et al. 2008 [33]	66.46	0	-	100.00	100.00	100.00	4	6.02 (2.259–16.037)	81.94	69.91	81.94
Beuer et al. 2009 [30]	68.99	2	2.89 (0.725–11.589)	91.30	85.51	71.01	1	1.45 (0.204–10.289)	95.65	92.75	95.65
Vult Von Steyern et al. 2005 [47]	40.00	0	-	100.00	100.00	100.00	3	7.50 (2.419–23.254)	77.50	62.50	77.50
Perry et al. 2012 [37]	32.00	0	-	100.00	100.00	100.00	2	6.25 (1.563–24.990)	81.25	68.75	81.25
Selz et al. 2015 [43]	36.00	0	-	100.00	100.00	100.00	19	13.89 (5.781–33.368)	58.33	30.56	58.33
Sailer et al. 2006 [31]	138.60	7	5.05 (2.408–10.594)	84.85	74.75	49.49	8	5.77 (2.887–11.542)	82.68	71.14	82.68
Naenni et al. 2015 [36]	116.00	0	-	100.00	100.00	100.00	30	10.34 (5.875–18.216)	68.97	48.28	68.97
Schmitt et al. 2009 [42]	77.00	0	-	100.00	100.00	100.00	3	3.90 (1.257–12.080)	88.31	80.52	88.31
Reich et al. 2014 [39] ^§^	138.87	2	1.44 (0.36–5.759)	95.68	92.80	85.60	5	3.60 (1.499–8.650)	89.20	82.00	89.20
Total	713.91	11					75				
Summary Estimate (95% Ci) ^#^			1.78 (1.435–2.216)	94.66	91.10	82.20		1.24 (0.932–1.661)	96.27	93.78	87.56
**Study and** **Year of** **Publication**	**Total FDP Exposure Time**	**Total No of Failures**	**Estimated Failure Rate Per 100 FDP Years (95% CI)**	**Estimated 3 Year Survival Rate**	**Estimated 5 Year Survival Rate**	**Estimated 10 Year Survival Rate**	**Total No. of Complications**	**Estimated Complication Rate (Per 100 FDP Years)**	**Estimated 3 Year Success Rate**	**Estimated 5 Year Success Rate**	**Estimated 10 Year Success Rate**
Lops et al. 2012 [35]	156.00	2	1.28 (0.321–5.126)	96.15	93.59	87.18	3	1.92 (0.620–5.963)	94.23	90.38	94.23
Raigrodski et al. 2012 [38]	94.00	3	3.19 (1.029–9.895)	90.43	84.04	68.09	4	4.26 (1.597–11.338)	87.23	78.72	87.23
Sorrentino et al. 2012 [45]	240.00	0	-	100.00	100.00	100.00	16	6.67 (4.084–10.882)	80.00	66.67	80.00
Burke et al. 2013	176.00	1	0.57 (0.08–4.034)	98.30	97.16	94.32	7	3.98 (1.896–8.343)	88.07	80.11	88.07
Solá-Ruiz et al. 2015 [44]	177.00	3	1.69 (0.547–5.255)	94.92	91.53	83.05	8	4.52 (2.260–9.038)	86.44	77.40	86.44
Ioannidis et al. 2016 [34]	365.10	3	0.82 (0.265–2.548)	97.53	95.89	91.78	23	6.30 (4.186–9.480)	81.10	68.50	81.10
Total	1208.10	12					61				
Summary Estimate (95% Ci) ^#^			2.07 (1.555–2.746)	93.80	89.67	79.33		1.08 (0.759–1.523)	96.77	94.62	89.25
**Study and** **Year of** **Publication**	**Total FDP Exposure time**	**Total No of Failures**	**Estimated Failure Rate Per 100 FDP Years (95% CI)**	**Estimated 3 Year Survival Rate**	**Estimated 5 Year Survival Rate**	**Estimated 10 Year Survival Rate**	**Total No. of Complications**	**Estimated Complication Rate (Per 100 FDP Years)**	**Estimated 3 Year Success Rate**	**Estimated 5 Year Success Rate**	**Estimated 10 Year Success Rate**
Rinke et al. 2018 [40]	695.27	24	3.45 (2.314–5.15)	89.64	82.74	65.48	50	7.19 (5.451–9.488)	78.43	64.04	78.43
Teichmann et al. 2018 [46]	213.50	1	0.47 (0.066–3.325)	98.59	97.66	95.32	9	4.22 (2.193–8.102)	87.35	78.92	87.35
Chaar et al. 2015 [32]	561.88	4	0.72 (0.267–1.897)	97.86	96.44	92.88	29	5.16 (3.587–7.427)	84.52	74.19	84.52
Total	1470.65	29					88				
Summary Estimate (95% Ci) ^#^			1.82 (1.716–1.935)	94.53	90.89	81.78		1.82 (1.479–2.244)	94.53	90.89	81.78

* Surface roughness was not considered as a complication in the statistics for 3-, 5-, and 10-year success; ^#^ Estimated with the Poisson-regression model (heterogeneity test based on Pearson goodness-of-fit was not significant (*p* > 0.05)); ^§^ Reich et al. [39] utilized lithium disilicate frameworks.

## Data Availability

Not applicable.
